# Identifying genetic causes and establishing a diagnostic approach for WES-negative pediatric population with neurodevelopmental disorder

**DOI:** 10.1038/s41431-026-02148-0

**Published:** 2026-06-23

**Authors:** Yeseul Kim, Joowon Jang, Kyeong Seon Ryu, Jong-Hee Chae, Jung Min Ko, Man Jin Kim, Seungbok Lee, Jangsup Moon, Jin Sook Lee, Hoyeon Lee, Sung Im Cho, Seung Won Chae, Hansol Lim, Hara Lim, Hobin Sung, Seonhoo Youn, Hyesu Lee, Jee-Soo Lee, Moon-Woo Seong

**Affiliations:** 1 Department of Laboratory Medicine, Seoul National University Hospital, Seoul National University College of Medicine, Seoul, Korea; 2 Department of Genomic Medicine, Seoul National University Hospital, Seoul, Korea; 3 Department of Pediatrics, Seoul National University Children’s Hospital, Seoul, Korea; 4 Department of Neurology, Seoul National University Hospital, Seoul, Korea; 5 Cancer Research Institute, Seoul National University College of Medicine, Seoul, Korea

**Keywords:** Genetics, Genetic testing

## Abstract

Global developmental delay (GDD) and intellectual disability (ID) are frequently caused by genetic factors, yet many patients remain undiagnosed even after whole exome sequencing (WES). This study aimed to apply Optical Genome Mapping (OGM) and Illumina Complete Long Reads (ICLR) in pediatric patients with unexplained GDD/ID after WES and propose a practical diagnostic strategy for clinical implementation. We conducted OGM and ICLR on 87 pediatric patients with unexplained GDD/ID despite prior WES. Discordant cases underwent further validation using gap-PCR or PacBio long-read sequencing. A minigene assay was also performed to confirm the pathogenicity of an intronic variant. Of the 87 patients, 6 were found to carry pathogenic or likely pathogenic variants, including 4 structural variants (SVs) and 2 single nucleotide variants (SNVs). OGM and ICLR provided additional diagnostic yields of 4.71% and 6.98%. OGM was effective in detecting complex rearrangements, whereas ICLR performed well in cases with overlapping structural variants. For all SV burden, ICLR detected 8 SVs (mean 0.09 ± 0.33 per sample), and OGM identified 8 SVs (mean 0.09 ± 0.29 per sample), showing comparable results. This study demonstrates the complementary utility of ICLR and OGM in detecting diverse classes of pathogenic variants in GDD/ID. ICLR was advantageous for detecting non-coding SNVs, as well as for providing accurate breakpoint resolution in SVs, while OGM was effective for complex rearrangements and repetitive regions. These findings support a stepwise diagnostic strategy in which ICLR may be considered as an early second-tier test for WES-negative GDD/ID cases.

## Introduction

Global developmental delay (GDD) and intellectual disability (ID) are two common features of neurodevelopmental disorders [1]. GDD is diagnosed in children under five with significant delays in at least two domains: motor, language, cognition, social interaction, or daily living. ID involves impaired intellectual and adaptive functioning and is diagnosed when such delays persist beyond five years [2]. GDD and ID have diverse underlying causes, including genetic and acquired factors, with genetic factors accounting for nearly half of all cases [3]. Down syndrome is the most prevalent chromosomal abnormality in GDD and ID, while Fragile X syndrome is the leading inherited monogenic cause [4].

Current guidelines recommend chromosomal microarray (CMA) and fragile X testing as first-tier investigations, followed by whole exome sequencing (WES) when initial tests are inconclusive [5]. However, WES yields a molecular diagnosis in only about 35% (ranging from 21 to 66%) of cases [6]. This limited diagnostic rate is largely attributable to technical limitations and restricted analytical scope, particularly in detecting structural variants (SVs), repeat expansions, non-coding variants, and variants located in sequencing-challenging regions. As a result, whole genome sequencing and genome-wide SV detection methods are being increasingly explored as complementary approaches [7]. Illumina’s Complete Long Reads whole genome sequencing (ICLR WGS) allows simultaneous short- and long-read sequencing within a single platform, featuring the capability to directly compare short and long read results from the same instrument [8]. In contrast, Bionano Optical Genome Mapping (OGM) analyzes ultra-long DNA molecules without the need for sequencing. Notably, OGM enables the identification of very large structural variants (more than 50 kb) and balanced translocations that may be challenging to detect even with long-read sequencing technologies [9].

In this study, we aimed to identify the genetic causes in a cohort of pediatric patients with unexplained GDD and ID who remained undiagnosed after WES, by applying WGS and long-read DNA-based genomic technologies. This study aimed to assess the diagnostic yield of two platforms and propose an expanded genetic evaluation strategy for neurodevelopmental disorders.

## Materials and methods

### Patient samples

During the study period, pediatric patients with global developmental delay or intellectual disability were identified from the Lee-Rare-Disease Project at Seoul National University Hospital. Patients with known non-genetic causes (e.g., congenital infection, trauma, malignancy, or metabolic disorders) or with positive CMA findings were excluded before defining the WES cohort. As a result, 213 pediatric patients with unexplained GDD/ID underwent trio WES at the Department of Molecular Diagnostics, Seoul National University Hospital between October 12, 2022, and January 11, 2024.

Among these 213 patients, WES provided a molecular diagnosis in 98 patients, whereas 115 remained undiagnosed. The present study focused on this WES-negative subset. Among the 115 WES-negative patients, 28 were excluded because suitable samples were unavailable or insufficient for downstream genomic testing. Consequently, 87 WES-negative GDD/ID probands were included for OGM and/or ICLR analysis. Among these 87 patients, CMA data were unavailable in 29 cases because CMA had not been performed, for example, due to financial constraints, or because external test reports were unavailable.

### Variant detection methods

#### Short-read WES

Genomic DNA was extracted from peripheral blood using the Chemagic 360 instrument (Perkin Elmer, Baesweiler, Germany), fragmented with a Covaris E220 focused ultrasonicator (Covaris, Woburn, MA, USA), and assessed using the TapeStation 4200 (Agilent Technologies, Santa Clara, CA, USA) and Qubit™ Flex Fluorometer (Thermo Fisher Scientific, Waltham, MA, USA). DNA fragments were captured using the SureSelect All Exon V8 kit (Agilent Technologies). A total of 500 ng DNA was used as the input and library preparation was performed using the SureSelect XT Target Enrichment Protocol. Sequencing was performed on the NovaSeq 6000 platform (Illumina, San Diego, CA, USA), generating 150 bp paired-end reads. FASTQ files were quality-checked using FastQC (Babraham Institute, Cambridge, MA, UK), aligned to hg19 using BWA v0.7.17, and processed with GATK v4.5.0.0 (Broad Institute, Cambridge, MA, USA). Variants were annotated using snpEff.

Variants with allele frequency >5% in gnomAD (https://gnomad.broadinstitute.org) were excluded. Remaining variants were prioritized based on phenotype relevance and absence of benign or likely benign classification in clinical databases such as ClinVar. Pathogenicity was evaluated using segregation data, population frequency, functional studies, and computational tools such as CADD, REVEL, and SpliceAI. Variants were interpretated followed by ACMG guidelines. The overall analytical workflow is illustrated in Supplementary Fig. 1, which outlines the application of ICLR and OGM to WES-negative cases.

#### Illumina complete long reads WGS

Long single-molecule DNA fragments are generated and then treated with enzymes that incorporate chemically modified nucleotides to create unique landmark patterns, following the Illumina Complete Long Read Prep protocol (Illumina, San Diego, CA, USA; Ref: 20089108, 200015544 v00). Library preparation, including the landmarking step, followed the Illumina DNA PCR-Free Library Prep Reference Guide (Illumina, San Diego, CA, USA; Ref: 20089108, 1000000086922 v03). Libraries were normalized to 2 nM and short-read sequencing was performed on both the marked and unmarked libraries using the NovaSeq 6000 (Illumina). The marked and unmarked sequencing data were combined to reconstruct highly accurate long reads using the Illumina DRAGEN platform, which detects landmark patterns via reference-based and k-mer-based strategies, followed by a de Bruijn graph-like assembly approach. Variants were confirmed by comparison to short-read data to exclude artificial landmark signals. In 86 samples, key quality metrics of ICLR-rendered long reads showed improvement compared to the short-read data, with error and substitution rates decreasing from 0.066 to 0.004 and from 0.0653 to 0.0025, respectively (Supplementary Table 1). ICLR reads had a mean coverage of 29× (vs. 255× in short reads), an N50 of 6229 bp. The fraction of bases with <10× coverage was 3.88% and 0.24% of reads had a mapping quality score of zero (MQ0). Short reads were aligned using DRAGEN module; long reads using a modified Minimap2. Small variants (including SNVs and indels) were called independently from each dataset with the DRAGEN small variant caller, merged into a VCF via a GIAB-trained ML model, and phased using the ICLR and modified WhatsHap. SVs were called using Sniffles2 (for long reads) and DRAGEN SV caller (for short reads), then merged into a unified VCF.

For SNVs, variants within 1497 GDD/ID-related genes (PanelApp, OMIM, Phenomizer) were retained for further analysis. The filtered SNV VCFs were then annotated using ANNOVAR. Because one aim of this study was to evaluate the diagnostic utility of genome sequencing beyond the coding regions typically captured by exome sequencing, we also assessed non-coding variants during variant interpretation. Coding variants were interpreted as in short-read WES; non-coding variants with SpliceAI ≥0.5 were retained and evaluated by ACMG guidelines. For SVs, the SV VCFs were annotated using AnnotSV v3.0.5. SVs ≥1 kb involving OMIM genes were selected. Recurrent SVs in ≥2 individuals were excluded. Only SVs involving coding-regions were retained. All filtered variants were manually reviewed using IGV to eliminate false calls. Pathogenicity was evaluated using DECIPHER, OMIM, and ACMG/ClinGen guidelines [10, 11]. Final candidate variants were reviewed for phenotype correlation and inheritance pattern.

#### Optical genome mapping

Whole blood samples stored at –80 °C were transported on ice and thawed at 37 °C for 2 minutes before DNA extraction. Ultra-high molecular weight (UHMW) DNA was isolated using manufacturer’s protocols (Bionano Genomics, San Diego, CA, USA), including isopropanol precipitation, and capture with Nanobind magnetic disks. DNA labeling was performed with 750 ng of UHMW DNA using Direct Labeling Enzyme 1 (DLE-1), targeting the 6-mer motif (CTTAAG) without cleaving the backbone. Labeled DNA was counterstained and loaded onto Saphyr G3.3 chips and imaged across nanochannels. Samples were run on the Saphyr System, targeting 400 Gbp per flowcell. Quality metrics included ~160× genome coverage, >70% mapping rate, N50 > 230 kbp, and label density of ~15 per 100 kbp. Data were analyzed using Bionano Solve software (v3.7 and v3.8) with two pipelines: a CNV pipeline for large, unbalanced aberrations and a de novo assembly pipeline for SV detection. In de novo assembly pipeline, pairwise comparison of all DNA molecules was performed to generate the initial consensus genome maps (cmap). SVs were detected by comparing the alignment of cmap and the GRCh37 assembly. Visualization and variant reporting were performed using Bionano Access v1.8.

SV filtering used the hg19 DLE-1 SV mask to exclude low-confidence regions. Confidence thresholds were: insertions/deletions = 0, inversions = 0.02, duplications = 0, translocations = 0.02, CNVs = 0.99. SVs were cross-referenced with a dataset of 285 Bionano control genomes. Only rare variants (<1%) with VAF ≥ 0.3 and overlapping OMIM genes were retained in Bionano Access v1.8. Variants ≥1 kb and within coding regions were prioritized; those found in ≥2 individuals or with ≥10 overlapping calls in DGV were excluded. The final pathogenicity assessment was performed in accordance with the methods described in the ICLR.

### Variant confirmation methods for discordant cases

#### Gap-PCR

Gap-PCR was performed in two discordant cases between ICLR and OGM to confirm a deletion and an inverted duplication in *KMT2C*, and an insertion in *NAA15*, using a Veriti Thermal Cycler (Applied Biosystems, USA). *KMT2C* deletion and inverted duplication was detected using standard PCR with standard Taq polymerase (Thermo Fisher Scientific), and *NAA15* insertion was detected via long-range PCR with LA Taq polymerase (Takara Bio Inc., Kusatsu, Shiga, Japan). Amplicons were resolved on 1.5% agarose gels, purified with ExoSAP-IT (Thermo Fisher Scientific), and sequenced with BigDye Terminator v3.1 and ABI 3730 DNA Analyzer. Sequence data were analyzed with Chromas and NCBI BLAST. For primer design, candidate regions were identified using BLAST, and primers were designed with Primer3Plus. *KMT2C* primer set 1 for detecting a deletion overlapping with an inverted duplication(F:5′-GCTCACCATGCTTTGCTGTC-3′; R:5′-CCAACGAGTTCCAGAGCCTT-3′) produced a 514 bp product in the patient only. The *NAA15* primer set(F:5′-TGCAGTGCATGTGGTTCCAA-3′; R:5′-AATGGCTTGCTGGGCAAAGA-3′) yielded a 9900 bp product in the patient and a 7212 bp product in the control, indicating a 2688 bp insertion. Primer specificity was verified using in silico PCR (UCSC Genome Browser, GRCh38/hg38).

#### Minigene assay

To evaluate the splicing impact of the *KCNJ6* deep intronic variant (c.946+3003 C > T), minigene constructs were generated using the pSPL3 exon trapping vector. Genomic DNA fragments (~500–600 bp) flanking the variant were PCR-amplified from the proband’s DNA using primers with restriction sites (Forward: 5′-ctgactgaGAATTCGGAGGTGTCTGGAAACTGGA-3′; Reverse: 5′-tcagtcagGGATCCGTCAATCAGCAAGGCCGTAG-3′) as described in previous study [12] and cloned into the pSPL3 vector. Wild-type and mutant constructs were transfected into HEK293T cells using Lipofectamine 3000 (Thermo Fisher Scientific). After 48 h, RNA was extracted with TRIzol and RT-PCR was performed using pSPL3-specific primers (Forward: 5′-TCTGAGTCACCTGGACAACC-3′; Reverse: 5′-ATCTCAGTGGTATTTGTGAGC-3′). PCR products were analyzed by agarose gel electrophoresis and confirmed by Sanger sequencing.

#### Pacbio SMRT

Genomic DNA was extracted from 300 µL of whole blood stored at –80 °C using the Wizard® HMW DNA Extraction Kit (Promega Corp., Madison, WI, USA). Short fragments were removed using the PacBio Short Read Eliminator Kit, and remaining high molecular weight (HMW) DNA was sheared to 15–20 kb using a Covaris g-TUBE. DNA cleanup, damage repair, A-tailing, and adapter ligation with barcoded SMRTbell adapters were performed, followed by nuclease treatment and size selection using AMPure PB beads to retain fragments >5 kb. Final libraries were prepared using the Revio SPRQ™ Polymerase Kit and sequenced on the PacBio Revio platform.

Raw reads were aligned to the UCSC hg38 genome using pbmm2 (v1.13.1). SNPs and indels were identified using DeepVariant (v1.5.0), and SVs were identified using pbsv (v2.9.0). Phasing was performed with HiPhase and WhatsHap (v2.1), and CNVs were analyzed with HiFiCNV (v1.0.0). Tandem repeat expansions were detected with TRGT (v1.0.0), and methylation was detected using pb-CpG-tools (v2.3.2). SNPs/indels were annotated with ANNOVAR, and SVs with AnnotSV (v3.4.2).

## Results

### Comprehensive diagnostic outcomes revealed by two genomic approaches

**Fig. 1:**
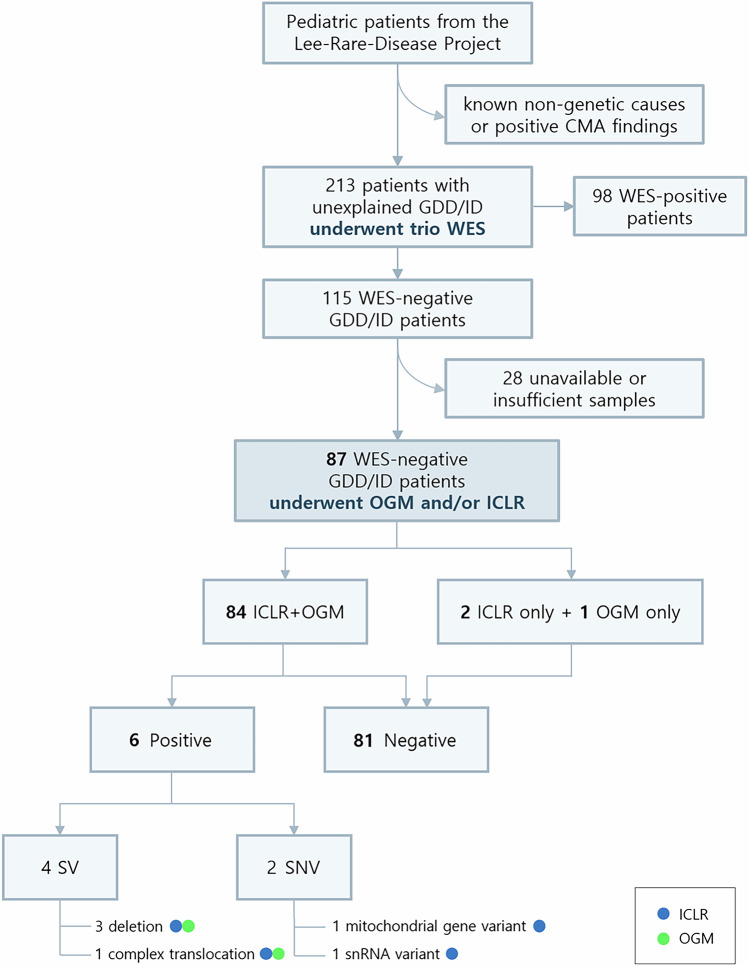
Study design and diagnostic outcomes in patients with unexplained GDD or ID. After excluding patients with known non-genetic causes or positive CMA findings, 213 pediatric patients with unexplained GDD/ID underwent trio WES. Among them, 98 received a molecular diagnosis by WES and 115 remained WES-negative. Of these, 87 had available samples and underwent downstream OGM and/or ICLR analysis. Among the 87 WES-negative probands, 84 underwent both ICLR and OGM, 2 underwent ICLR only, and 1 underwent OGM only. Pathogenic or likely pathogenic variants were identified in 6 cases: 4 structural variants and 2 SNVs. For each of the six diagnostic variants, a circular icon is placed adjacent to the variants to indicate the detecting platform: blue circles denote variants detected by ICLR, and green circles denote variants detected by OGM. WES whole exome sequencing, CMA chromosomal microarray, GDD global developmental delay, ID intellectual disability, ICLR Illumina’s Complete Long Reads, OGM Optical Genome Mapping, SV structural variant, SNV single nucleotide variant.

We performed two genomic assays—ICLR and OGM—in 87 WES-negative patients with unexplained GDD or ID. Due to insufficient DNA quantity, two samples underwent only ICLR. One sample underwent only OGM, and its results were compared with external whole genome sequencing (WGS) data. Pathogenic or likely pathogenic variants were identified in 6 cases (Fig. 1). In all six, ICLR identified the variants of interest; OGM only identified four of six variants. Four were structural variants (SVs): 3 deletions and 1 deletion with complex rearrangement. The four SVs (case 1, 2, 3, and 4) were detected by both ICLR and OGM, while in case 3 OGM resolved inter/intrachromosomal translocations in addition to the deletion, whereas ICLR detected only intrachromosomal events. All SVs detected by ICLR were also visualized in the short-read Illumina BAM files. The remaining 2 were SNVs not typically captured by WES, including a mitochondrial variant and a variant in the snRNA gene *RNU4-2*, detected only by ICLR (cases 5 and 6). Details of the positive cases and their genetic and clinical features are provided (Table 1).

**Table 1:** Summary of genetic findings and clinical features in 6 diagnosed cases.

Information		Case 1	Case 2	Case 3	Case 4	Case 5	Case 6
Sex/Age		F/2	M/0	M/4	M/9	M/12	M/1
CMA result		NA	normal	NA	normal	NA	normal
Results from this study	Variant type	Structural variant	Structural variant	Structural variant	Structural variant	mitochondrial gene variant	snRNA variant
	Included gene	*SMARCC2*	*ATRX*	*ZEB2*	*KANSL1*	*MT-TL1*	*RNU4-2*
	ICLR results (hg38)	3.3 kb Deletion	73 kb Deletion	1.6 Mb deletion	5.6 kb deletion	m.3243 A > G	n.64_65insT
	OGM results	3.3 kb Deletion	73 kb Deletion	1.6 Mb deletion with complex translocation	5.6 kb deletion	not detected	not detected
phenotype	Growth					short stature	short stature
	Head & neck		Microcephaly Hypertelorism Laryngomalacia		Strabismus		Microcephaly
	Abdomen		Anal stenosis				Feeding difficulties
	Neurologic (central nervous system)	Global developmental delay	Global developmental delay	Global developmental delay	Global developmental delay Intellectual disability Hypoplasia of the corpus callosum Seizures	Intellectual disability	Global developmental delay Gray matter heterotopia
	Neurologic (peripheral nervous system)	Hypotonia	Hypotonia				
	Neurologic (Behavioral Psychiatric manifestation)			Epileptic aura	Autistic behavior ADHD		
	Others			Vesicoureteral reflux	Cryptorchidism Pectus excavatum	Renal hypoplasia Hypertrichosis	

*OGM* Optical Genome Mapping, *ICLR* Illumina’s Complete Long Reads, *CMA* chromosomal microarray.

We evaluated the diagnostic yield of WES in 213 patients with unexplained GDD/ID and the additional yields of OGM and ICLR in the 87 WES-negative patients who underwent downstream genomic testing (Table 2). WES solved 98 of the 213 cases (46.01%). Of the 115 WES-negative cases, 87 underwent OGM and/or ICLR, whereas 28 were excluded because of unavailable or insufficient samples. OGM identified pathogenic SVs in 4 of 85 tested cases (4.71%), while ICLR detected 4 SVs and 2 SNVs in 6 of 86 tested cases (6.98%), indicating a slightly higher additional diagnostic yield for ICLR compared with OGM.

**Table 2:** Diagnostic yield of WES and additional genomic tests in GDD/ID patients.

Method	Total diagnosed	Diagnostic yield	Additionally diagnosed	Additional diagnostic yield
WES	98/213*	46.01%		
OGM			4/85	4.71%
ICLR (SV only)			4/86	4.65%
ICLR (SV + SNV)			6/86	6.98%

*Among the 213 cases analyzed, after excluding 98 diagnosed cases, a total of 115 negative cases remained; of these, 28 were not further evaluated due to insufficient sample quantity, and thus only the remaining 87 cases were subjected to subsequent OGM and ICLR experiments.

*WES* whole exome sequencing, *GDD* global developmental delay, *ID* intellectual disability, *OGM* Optical Genome Mapping, *ICLR* Illumina’s Complete Long Reads, *SV* structural variant, *SNV* single nucleotide variant.

### Case descriptions

**Fig. 2:**
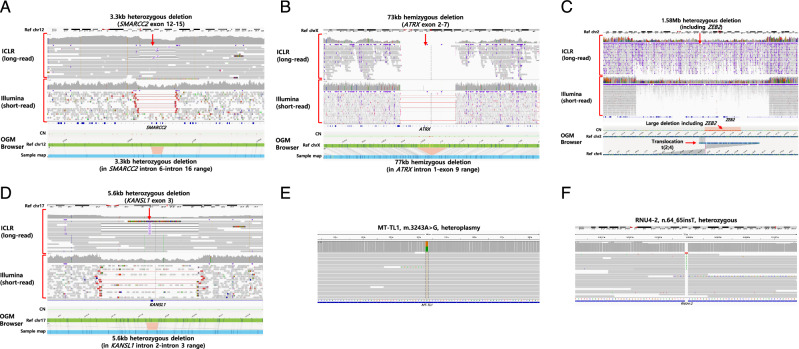
Overview of six positive cases: variant detection by ICLR and OGM and validation using Gap-PCR. **A** Case 1 (GL00109P): Heterozygous 3.3 kb deletion (NC_000012.12:g.56176903_56180201del, GRCh38) involving *SMARCC2* exons 12–15 detected by both ICLR and OGM in a female patient with GDD and hypotonia. **B** Case 2 (GL00355P): Hemizygous 73 kb deletion (NC_000023.11:g.77685462_77758609del, GRCh38) involving *ATRX* exons 2–9 identified by both ICLR and OGM in a male patient with GDD, hypotonia, and dysmorphic features. **C** Case 3 (GL00609P): A 1.6 Mb deletion (NC_000002.12:g.143349256_144926199del, GRCh38) involving *ZEB2* identified by both platforms, with an interchromosomal translocation additionally revealed by OGM in a male patient with GDD and epileptic aura. **D** Case 4 (GL00676P): Heterozygous 5.6 kb deletion (NC_000017.11:g.46091685_46097343del, GRCh38) involving *KANSL1* exon 3 detected by both platforms in a syndromic male patient with GDD and seizures; filtered by OGM due to SV mask region. **E** Case 5 (GL00206P): Heteroplasmic m.3243 A > G variant (NC_012920.1: n.14 A > G) in *MT-TL1* identified by ICLR in a male patient with ID and renal anomalies; variant known to cause MELAS. **F** Case 6 (GL00372P): Pathogenic SNV (NR_003137.3:n.64_65insT) in *RNU4-2* detected by ICLR in a male patient with GDD and brain malformation; associated with disrupted spliceosome function. ICLR Illumina’s Complete Long Reads, OGM Optical Genome Mapping, CN copy number, GDD global developmental delay, SV structural variant, SNV single nucleotide variant, ID intellectual disability, MELAS mitochondrial encephalomyopathy, lactic acidosis, and stroke-like episodes.

Case 1 (GL00109P): A 2-year-old girl with GDD and hypotonia had a 3.3 kb heterozygous deletion (NC_000012.12:g.56176903_56180201del, GRCh38) encompassing *SMARCC2* exons 12–15, detected by both ICLR and OGM (Fig. 2A). Although not yet listed in dosage sensitivity databases, *SMARCC2* is associated with autosomal dominant Coffin-Siris syndrome 8 (#MIM 618362) and shows high constraint (pLI = 1). Loss-of-function variants in *SMARCC2* have been reported in affected individuals [13]. The variant was classified as likely pathogenic based on ACMG guidelines and prior literature [14].

Case 2 (GL00355P): A 1-year-old boy with GDD, hypotonia, microcephaly, hypertelorism, and laryngomalacia had a 73 kb hemizygous deletion (NC_000023.11:g.77685462_77758609del, GRCh38) spanning *ATRX* exon 2–9, identified by both ICLR and OGM (Fig. 2B). *ATRX* is associated with X-linked intellectual disability-hypotonic facies syndrome (#MIM 309580) and shows high intolerance to haploinsufficiency (pLI = 1, HI = 3). This variant was classified as pathogenic.

Case 3 (GL00609P): A 4-year-old boy with GDD, epileptic aura, and vesicoureteral reflux had a 1.6 Mb heterozygous deletion (NC_000002.12:g.143349256_144926199del, GRCh38) involving *ZEB2*, detected by both ICLR and OGM (Fig. 2C). *ZEB2* is associated with autosomal dominant Mowat-Wilson syndrome (#MIM 235730) (pLI = 1, HI = 3). This variant was classified as pathogenic and considered the causal variant explaining the patient’s phenotype. To further characterize the structural architecture, we examined additional structural signals detected by OGM and ICLR. Further inspection of OGM SV data, together with soft-clipped regions observed in ICLR, enabled reconstruction of a genomic map delineating both intra- and interchromosomal rearrangement breakpoints (Supplementary Fig. 2). OGM revealed a complex rearrangement involving an inverted insertion from chromosome 4 into chromosome 2, resulting in an unbalanced translocation with a partial deletion. These additional breakpoints did not involve protein-coding genes with known gene-phenotype associations. ICLR captured the associated deletions and duplications but did not resolve their structural configuration, highlighting the advantage of OGM in resolving complex chromosomal rearrangements.

Case 4 (GL00676P): A 9-year-old boy with GDD, ID, seizures, autism, ADHD, and corpus callosum hypoplasia had a 5.6 kb heterozygous deletion (NC_000017.11:g.46091685_46097343del, GRCh38) of *KANSL1* exon 3. The variant was initially detected by ICLR and was also present in the OGM genome maps; however, it was excluded from the default OGM SV call set because it was masked by the DLE-1 SV filter used in the analysis pipeline. This filter is designed to reduce false-positive calls in regions with suboptimal labeling patterns associated with the DLE-1 enzyme motif. The deletion was confirmed upon manual inspection of the OGM data (Fig. 2D). *KANSL1* is linked to autosomal dominant Koolen-De Vries syndrome (#MIM 610443) (pLI = 1, HI = 3). This variant was classified as likely pathogenic.

Case 5 (GL00206P): A 12-year-old boy with ID, short stature, renal hypoplasia, and hypertrichosis had a heteroplasmic m.3243 A > G variant (NC_012920.1: n.14 A > G) in *MT-TL1*, detected by ICLR (Fig. 2E). This variant is linked to MELAS in ~80% of cases [15]. Some clinical features were not fully explained by MELAS, warranting further investigation.

Case 6 (GL00372P): A 1-year-old boy with GDD, gray matter heterotopia, microcephaly, and feeding difficulties had a heterozygous insertion (NR_003137.3:n.64_65insT) in *RNU4-2*, detected by ICLR (Fig. 2F). This variant is a recurrent pathogenic mutation known to disrupt spliceosome function and cause autosomal dominant ReNU syndrome syndrome (#MIM 620851) [16].

### Functional validation of the KCNJ6 intronic variant (minigene assay)

For the evaluation of intronic variants, candidates with SpliceAI delta scores ≥0.5—the recommended threshold indicating a high probability of splice alteration—were initially filtered [17]. Only variants located in OMIM genes relevant to the patient’s phenotype were retained, and minigene assays were performed for variants that could plausibly be classified as likely pathogenic based on functional evidence.

**Fig. 3:**
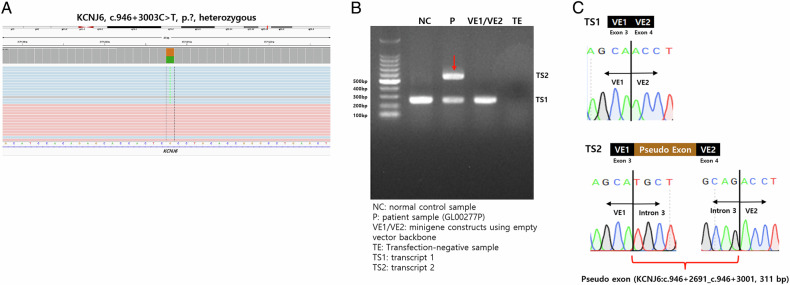
Functional validation of an intronic variant in *KCNJ6* using minigene assay in case GL00277P. **A** Deep intronic SNV (NM_002240.5:c.946+3003 C > T) in *KCNJ6* detected by ICLR in a female patient with GDD in IGV. **B** RT-PCR and gel electrophoresis revealed two transcripts: a 263 bp wild-type (TS1) consist of exon 3 and exon 4 and a 574 bp aberrant isoform (TS2) containing a 311 bp pseudoexon. In the patient sample, the TS2 band showed greater intensity, suggesting predominant expression of the aberrant transcript. **C** Sanger sequencing of TS1 confirmed canonical splicing between exon 3 and exon 4, while TS2 demonstrated aberrant inclusion of a cryptic 311 bp pseudoexon (NM_002240.5:c.946+2691_c.946+3001), consistent with pseudoexonization driven by the intronic variant. VE1 vector construct containing exon 3 of *KCNJ6*, VE2 vector construct containing exon 4 of *KCNJ6*.

In Case GL00277P, a 1-year-old girl with global developmental delay, periventricular leukomalacia, umbilical hernia, and facial dysmorphisms harbored a heterozygous deep intronic SNV (NM_002240.5:c.946+3003 C > T) in *KCNJ6* with a SpliceAI score of 0.56, detected by ICLR (Fig. 3A). *KCNJ6* is associated with autosomal dominant Keppen-Lubinsky syndrome (#MIM 614098). Given the elevated SpliceAI score and partial phenotypic overlap, a minigene assay was performed. The assay revealed a wild-type transcript (263 bp) and an aberrant transcript (574 bp) containing a 311 bp pseudoexon between exons 3 and 4 (Fig. 3B). Predominance of the aberrant isoform indicated splice disruption, although the presence of both transcripts suggested an incomplete splicing defect [17].

Currently, pathogenic variation in *KCNJ6* have been limited to in-frame deletions and missense variants, with no reported intronic or pseudoexon-forming pathogenic variants [18]. Given the absence of established haploinsufficiency evidence (HI score = 0) and limited support for a loss-of-function mechanism, the variant is classified as a VUS with functional evidence supportive of splice alteration (PM2, PP3).

### Discordant findings between optical mapping and long-read sequencing: validation for OGM-only or ICLR-only SVs in three suspected cases

During the evaluation of structural variants identified in the diagnostic workflow, three variants were detected by only one platform, resulting in discordant findings between OGM and ICLR. To further investigate these discordant findings, additional validation was performed in the three corresponding cases. In case GL00612P, OGM identified an 11.6 kb heterozygous deletion (OGM[GRCh37] 16q22.1(67599731_67640501)x1) spanning *CTCF* exon2 not detected by ICLR (Supplementary Fig. 3A). Subsequent PacBio long-read sequencing also failed to detect this variant, supporting ICLR result and indicating the OGM call was a false positive.

In case GL00536P, OGM detected a 2.7 kb heterozygous insertion (OGM[GRCh37] ins(4;?)(q31.1;?)(140281756_140288468;?)) affecting exon 13 to intron 14 of *NAA15* not identified by ICLR (Supplementary Fig. 3B). Gap-PCR showed two bands, consistent with a heterozygous insertion (Supplementary Fig. 3C). PacBio sequencing detected a 2.7 kb heterozygous duplication in intron 14, flanked by AluS elements. Comparison against population SV resources, including dbVar (nssv17664666) and the corresponding UCSC Genome Browser tracks (CoLoRSdb), indicates that this duplication is a common variant present in the general population. Although the OGM call was validated, the variant was interpreted as likely benign, and the case was interpreted as negative.

In case GL00417P, 2.4 Mb heterozygous deletion (NC_000007.14:g.149878789_152361926del, GRCh38) from exon 2 of *KMT2C* was detected by ICLR only (Supplementary Fig. 4A) The deletion was validated by gap-PCR and Sanger sequencing (Supplementary Fig. 4B, C). PacBio sequencing confirmed the deletion structure within the inverted duplication (Supplementary Fig. 4D–G). As the copy number of the structurally intact *KMT2C* gene was determined to be two, the case was interpreted as negative. OGM identified only an inversion, but they were excluded due to high frequency in control samples (Supplemental Table 2).

### Comparison of SV detection counts and methodological differences between OGM and ICLR

We compared the SV call burden and the number of clinically relevant SVs detected by OGM and ICLR in 87 samples. To account for platform-specific differences, we quantified the mean SV count per sample before and after sequential filtering. OGM produced an average of 31,041 raw SV calls per sample, whereas ICLR generated 4740 raw calls (Table 3). After stage 1 filtering—including shared criteria (SV size >1 kb, overlap with OMIM gene CDS with dominant inheritance, exclusion of recurrent SVs) and platform-specific criterion (DGV/DLE-1 masking for OGM; ACMG LB/B exclusion for ICLR)—the mean number of retained SVs decreased to 3.62 per sample for OGM and 2.99 for ICLR.

**Table 3:** Summary of structural variant (SV) counts across sequential filtering stages for OGM and ICLR.

Platform	Measure	Raw SV calls	Stage 1: Primary filters applied	Stage 2: DS-gene subset	Stage 3: Large SVs (≥20 kb)
OGM (*n* = 86)	Total count	26,69,551	311	103	8
	Per-sample mean	31,041.29	3.62	1.2	0.09
ICLR (*n* = 85)	Total count	4,02,875	254	40	8
	Per-sample mean	4,739.71	2.99	0.47	0.09

* Primary filtering included: (i) size >1 kb, (ii) overlap with autosomal dominant OMIM genes, and (iii) exclusion of recurrent events (>2 samples). Platform-specific rules were additionally applied (DGV/DLE-1 masking for OGM; ACMG class 1–2 exclusion for ICLR).

*OGM* Optical Genome Mapping, *ICLR* Illumina’s Complete Long Reads, *SV* structural variant, *DS* dosage-sensitive.

Further stage 2 filtering using 843 dosage-sensitive genes reduced the mean SV count to 1.20 (OGM) and 0.47 (ICLR) per sample. When restricting to SVs ≥20 kb in stage 3, both platforms converged to a similar residual burden (0.09 SVs per sample), yielding eight clinically relevant candidate SVs detected by OGM (*n* = 85) and ICLR (*n* = 86). Among these eight variants in ICLR, two corresponded to the pathogenic SVs identified in Case 2 and Case 3. One variant corresponded to the discordant call between OGM and ICLR (Case GL00417P), whereas the remaining five were excluded as causal variants and considered platform-specific false positives, as the affected genes or genomic regions were not consistent with the patients’ clinical phenotypes.

## Discussion

In our evaluation of unexplained GDD/ID cases, ICLR showed a higher additional diagnostic yield (6.98%) than OGM (4.71%). Notably, when considering only SV diagnoses, ICLR showed similar yield (4.65%), suggesting its strength lies not only in SNV detection but also in identifying SVs. This finding is consistent with previous studies reporting the strength of long-read WGS in capturing both balanced and unbalanced SVs [9]. However, the additional diagnostic yield from ICLR did not reach the upper range reported in prior from genome sequencing (9–15%) and long-read sequencing studies (7–17%), possibly due to our stringent filtering strategy and the use of short-read-based reconstruction of ICLR [19–21]. All SVs identified by ICLR showed corresponding split-read in short-read BAM files. This indicates that ICLR leveraged short-read–derived information during synthetic long-read reconstruction, which may partly limit its ability to resolve complex SVs compared with native long-read sequencing. Similarly, the additional diagnostic yield of OGM was lower than previously reported (10.6%) in WES-negative neurodevelopmental cases [22].

ICLR identified a deep intronic SNV *in KCNJ6* (case GL00277P), predicted to cause aberrant splicing. A minigene assay confirmed cryptic exonization between exons 3 and 4, supporting splice disruption. The presence of both wild-type and aberrant transcripts suggests an incomplete splicing defect. Although this provides functional support for splice alteration, clinical interpretation remains uncertain because KCNJ6 lacks established haploinsufficiency or loss-of-function disease mechanisms; therefore, the variant does not meet criteria for pathogenic or likely pathogenic classification. However, this case highlights the value of combining long-read sequencing with functional assays to interpret intronic variants. Additional in vivo investigations, including quantitative transcript analysis and assessment of downstream effects on protein function, can be necessary to clarify the pathogenic relevance.

We observed several instances where SVs were detected by only one platform, requiring orthogonal validation. In Case GL00536P, OGM detected a heterozygous insertion in *NAA15* not captured by ICLR. The failure of ICLR reflects mapping challenges in Alu-rich regions, as ICLR relies on short-read-based enrichment and may filter or misalign repeat elements. OGM, relying on high-molecular-weight DNA and label-based mapping, is less susceptible to sequence repetitiveness. As population-wide SV reference datasets expand, recurrent benign CNVs will be more readily recognized and excluded during variant prioritization. In Case GL00417P, PacBio sequencing validated the deletion within an inverted duplication, demonstrating PacBio’s strength in resolving complex, overlapping SVs, whereas ICLR showed ambiguous clipped sequences. Such variants may be missed by OGM due to sparse label density, dominance of large signals, and limited resolution of split molecule alignments.

Taken together, ICLR enables base-pair-level breakpoint refinement and improves detection of deep intronic and noncoding variants that are typically missed by ES or short-read GS. While ES is effective for coding SNVs/indels and short-read GS offers genome-wide coverage, both are limited in resolving SVs within repetitive or structurally complex regions. OGM excels in detecting large and complex rearrangements, including multi-breakpoint inversions and inter- and intrachromosomal translocations, through its optical mapping of ultra–high-molecular-weight DNA. This enables visualization of SVs that are challenging to resolve with sequencing-based methods, although OGM cannot capture small sequence-level changes. PacBio HiFi GS offers the most comprehensive long-read solution for SVs, repeat expansions, and phasing, but requires high-molecular-weight DNA and incurs higher per-sample cost, which may limit broader clinical implementation. A comparative summary of ES, short-read GS, PacBio HiFi GS, ICLR, and OGM is provided in Supplementary Table 3.

To compare structural variant calls between OGM and ICLR under standardized filtering conditions, we applied a sequential filtering framework. This analysis was performed independently of the initial diagnostic interpretation used to identify pathogenic variants. Using this filtering approach, eight candidate SVs of potential clinical relevance were identified across the analyzed samples. Notably, ICLR initially produced a substantial number of very large (≥10 Mb) recurrent SVs, which were removed during filtering as they were consistent with platform-specific artifacts. After exclusion of these events, the number of ICLR candidate SVs converged with that of OGM. Among the 8 retained SVs per platform, only three SVs were validated as true pathogenic variants, while the remaining five represented platform-specific false positives. We report the validated false-positive burden (≈0.06 spurious SVs per sample) and the call-reduction rate (>99.9% of raw calls discarded), noting that the latter reflects the combined removal of technical artifacts, benign or recurrent population variants, and SVs without plausible pathogenic effect under our filtering framework.

Our study highlights the clinical utility of both OGM and ICLR in evaluating unexplained GDD/ID. While combining both platforms offers the most comprehensive variant detection, a stepwise approach may be more practical in clinical settings. Given its higher diagnostic yield and ability to detect both SNVs and SVs, ICLR may serve as a first-line test, with OGM as a follow-up to detect large or complex rearrangements missed by sequencing-based methods. However, ICLR has limitations compared to true single-molecule long-read platforms like PacBio, as it relies on short-read-based sequencing and amplification. Unlike PacBio, which enables analysis of full-length RNA isoforms and methylation without amplification, ICLR lacks the capacity for integrated analyses of transcript structure and epigenetic regulation.

Several limitations of this study should be acknowledged. The modest sample size may limit generalizability, and the absence of RNA sequencing restricted functional assessment of intronic or regulatory variants. In addition, because ICLR relies on amplification and short-read backbone, the results should not be generalized to all long-read technologies. Further analysis is planned to explore unresolved cases. For SVs, we will focus on variants in OMIM genes affecting regulatory or untranslated regions and investigate misexpression caused by SVs in inactive or unannotated genomic regions, which may expand our understanding of gene regulation in neurodevelopmental disorders. For SNVs, the scope will expand beyond the initial 1,497 GDD/ID genes, and SpliceAI thresholds will be lowered to capture variants with moderate splicing effects. Genome-wide constraint metrics will also be incorporated to identify potentially novel disease-associated genes beyond those currently listed in OMIM.

In summary, this study demonstrates the complementary strengths of OGM and ICLR in identifying pathogenic variants in patients with unexplained GDD/ID. ICLR showed slightly higher diagnostic yield, due to its ability to detect intronic or small structural variants and showed superior breakpoint resolution. In contrast, OGM excelled in resolving complex, large-scale, and balanced rearrangements, reinforcing its utility in assessing overall genome architecture. While each platform has specific limitations, their combined use enhances diagnostic sensitivity and breadth, supporting a multimodal strategy in genetic diagnostics. Our findings suggest that combining OGM and ICLR offers a practical and comprehensive alternative to traditional WES-based diagnostics. Integrating both technologies into clinical workflows could enhance diagnostic accuracy and broaden the spectrum of detectable variant types in neurodevelopmental cases.

## Supplementary information

Supplemental materials

## Data Availability

All data generated or analyzed during this study are included in this published article and its supplementary information files.
