# Supplementary material for: Identifying genetic causes and establishing a diagnostic approach for WES-negative pediatric population with neurodevelopmental disorder
Source: Eur J Hum Genet. 2026 Jun 23;34(8):1156–64. doi: 10.1038/s41431-026-02148-0 (PMC13424310; doi:10.1038/s41431-026-02148-0)
Supplement: Supplementary file 1 — Supplemental materials [file 41431_2026_2148_MOESM1_ESM.pdf]

Supplemental Table 1. ICLR quality metrics in 86 samples

Metrics	Short read sequencing (Illumina)	Long read sequencing (ICLR rendered)
Mean coverage	255	29
Median coverage	234	29
Error rate (%)	0.066	0.004
Substitution rate (%)	0.0653	0.0025
N50 (read length) (bp)		6,229
Fraction of bases with coverage <10x (%)		0.0387
Number of reads		17,383,182
Number of MQ0 reads		42310.3 (0.24% of total reads)

Supplemental Table 2. Summary of genetic findings and clinical features in 2 discordant(benign) Cases

Information		Case GL00612P	Case GL00417P
Sex/Age		F/1	M/12
CMA result		normal	arr7q36.1x3(benign)
Results from this study	Variant type	Structural variant	Structural variant
	Included gene	*CTCF*	*KMT2C*
	ICLR results (hg38)	not detected	2.4Mb deletion
	OGM results	11kb deletion	not detected
phenotype	Growth		
	Head & neck		Exotropia
	Abdomen	Feeding difficulties in infancy	
	Neurologic (central nervous system)	Global developmental delay	Global developmental delay<br>Intellectual disability<br>Seizures
	Neurologic (peripheral nervous system)	Hypotonia	
	Others	Preterm birth<br>Neonatal respiratory distress	Obesity

Abbreviation: OGM, Optical Genome Mapping; ICLR, Illumina′s Complete Long Reads; CMA, chromosomal microarray

Supplemental Table 3. Comparative strengths and limitations of major genomic technologies (ES, GS, PacBio HiFi, OGM, ICLR)

Technology	Strengths	Limitations	Variant classes best detected
Exome sequencing (ES)	• Cost-effective<br>• High depth for coding SNVs/INDELs<br>• Widely standardized in clinics	• Limited to exons<br>• Poor SV detection<br>• Cannot detect TREs or resolve complex loci	SNVs/INDELs in coding regions
Short-read genome sequencing (SR-GS)	• Genome-wide<br>• Moderate detection of CNVs/SVs<br>• High throughput, low cost	• poor in repetitive / high-similarity regions<br>• Misses many SVs, TREs, imprinting defects<br>• No methylation info	SNVs/INDELs, some CNVs
PacBio HiFi long-read sequencing (LR-GS)	• Best per-base accuracy among LRS<br>• Excellent SV detection (3–5× more than SR-GS)<br>• Detects TREs, methylation, phasing, pseudogene-rich loci<br>• Enables de novo assemblies	• Higher cost than SR-GS<br>• Requires high-molecular-weight DNA<br>• Lower throughput	SVs, TREs, complex loci, phasing, methylation
Illumina Complete Long Reads (ICLR)	• Long molecule phasing + Illumina accuracy<br>• Better mapping in high-similarity regions than SR-GS	• Generates platform-specific artifacts (extra-large recurrent SVs) requiring filtering<br>• Underdeveloped TRE detection compared to PacBio<br>• No methylation	SVs, phasing, complex loci (improved vs SR-GS)
Optical genome mapping (OGM)	• Highly sensitive for large SVs (>500 bp–Mb)<br>• Detects balanced or complex rearrangements well than GS<br>• Accurate long-range structure visualization<br>• Less affected by repetitive genomic sequences compared to ICLR	• Cannot detect SNVs/INDELs<br>• Limited detection of small SVs (~ <500 bp)<br>• No methylation or sequence-level resolution<br>• Boundary definition based on reference label patterns may cause misannotation of variant context (e.g., misclassifying non-coding as coding region SV)	Large SVs, balanced or complex re arrangements

Abbreviation: ES, exome sequencing; srGS, short-read genome sequencing; lrGS, long-read genome sequencing; OGM, optical genome mapping; ICLR, Illumina Complete Long Reads; SV, structural variant; SNV, single nucleotide variant; indel, insertion/deletion; TRE, tandem repeat expansion; CNV, copy number variant;

Supplemental Table 4. ACMG classification and criteria for six positive cases described in this study

Case	Chr	Start	Stop	Variant Type	Zygosity	Size	Notation	Gene affected	Predicted impact	ACMG criteria	Classification	Gene_count	Gene_name
GL00109P	chr12	56176903	56180201	deletion	heterozygous	3.3kb	NC_000012.12:g.56176903_56180201del, GRCh38	SMARCC2	Deletes exon 12-15 of SMARCC2; predicted to leat to a frameshift (loss-of-function)	PVS1,PM2,PP4 (2015 ACMG standards, 2018 update for PVS1)	Likely pathogenic	1	SMARCC2
GL00355P	chrX	77685462	77758609	deletion	hemizygous	73kb	NC_000023.11:g.77685462_77758609del, GRCh38	ATRX	Deletes part of the second to last exon; predicted to leat to a frameshift (loss-of-function)	score>0.99 (2020 ACMG/ClinGen CNV standards)	Pathogenic	1	ATRX
GL00609P	chr2	143349256	144926199	deletion	heterozygous	1.6Mb	NC_000002.12:g.143349256_144926199del, GRCh38	ZEB2	Deletes whole gene	score>0.99 (2020 ACMG/ClinGen CNV standards)	Pathogenic	3	ARHGAP15;GTDC1;ZEB2
GL00676P	chr17	46091685	46097343	deletion	heterozygous	5.6kb	NC_000017.11:g.46091685_46097343del, GRCh38	KANSL1	Deletes exon 3 of KANSL1; predicted to leat to a frameshift (loss-of-function)	PVS1,PM2,PP4 (ACMG 2015 standards, 2018 for PVS1)	Likely pathogenic	1	KANSL1
GL00206P	chrM	3243	3243	SNV	heteroplasmy	.	NC_012920.1: n.14A>G	MT-TL1		PS4_Strong, PS3_Strong, PP1_Strong, PM2, PM1, PP3 (2020 ACMG/AMP standards for mitochondrial DNA variant)	Pathogenic	1	MT-TL1
GL00372P	chr17	.	.	1bp insertion	heterozygous	.	NR_003137.3:n.64_65insT	RNU4-2		PS4_Strong, PS3_Strong, PS2_Strong, PM2, PP4	Pathogenic	1	RNU4-2

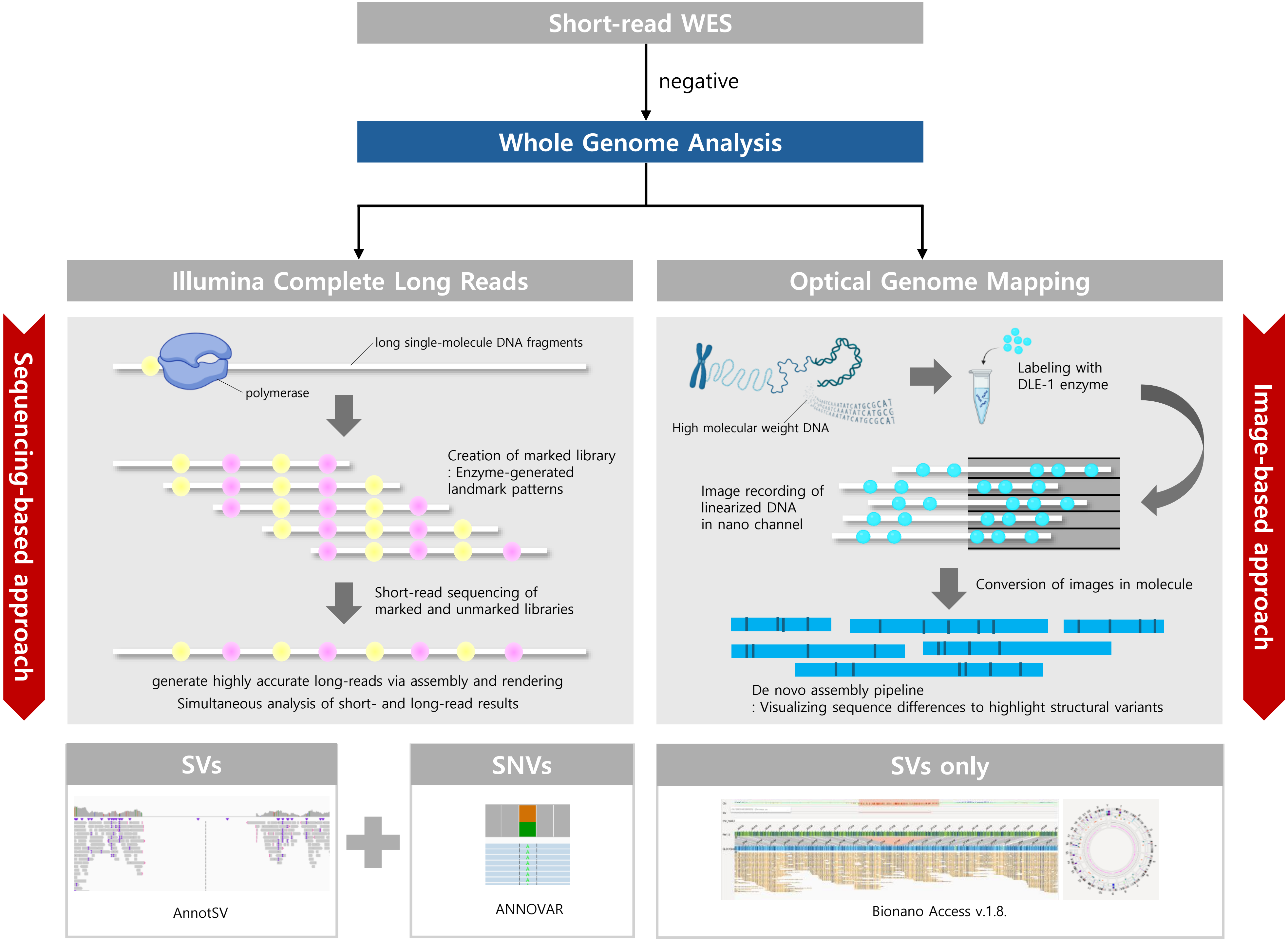

Short-read WES
negative
Whole Genome Analysis
Illumina Complete Long Reads
Optical Genome Mapping
Sequencing-based approach
long single-molecule DNA fragments
polymerase
Creation of marked library
: Enzyme-generated
landmark patterns
Short-read sequencing of
marked and unmarked libraries
generate highly accurate long-reads via assembly and rendering
Simultaneous analysis of short- and long-read results
High molecular weight DNA
Labeling with
DLE-1 enzyme
Image recording of
linearized DNA
in nano channel
Conversion of images in molecule
De novo assembly pipeline
: Visualizing sequence differences to highlight structural variants
Image-based approach
SVs
AnnotSV
SNVs
ANNOVAR
SVs only
Bionano Access v.1.8.

Supplemental Figure 1. Schematic overview of the analytical methods of this study
This figure illustrates two whole-genome analysis methods applied to WES-negative samples: Illumina Complete Long Reads (ICLR) and Optical Genome Mapping (OGM). ICLR is a sequencing-based approach in which long single-molecule DNA fragments are first labeled with polymerase-generated landmark patterns. Short-read sequencing is then performed as in conventional Illumina workflows, and the shared marks are used to assemble and rendering library to generate highly accurate long-reads. After mark removal, a second round of sequencing is performed on the long fragments, followed by variant calling. Final variant analysis is derived from the simultaneous integration of short- and long-read data, with SVs annotated by AnnotSV and SNVs by ANNOVAR.
OGM is an image-based approach that begins with the extraction of high-molecular-weight DNA fragments (>150 kb). DNA is labeled at 6-mer recognition sites (CTTAAG) using the DLE-1 enzyme, linearized, and loaded onto the Saphyr G3.3 chip for imaging in nanochannels. Structural variants and CNVs are subsequently detected by comparing de novo assemblies with reference sequences and visualized as differences in labeling patterns. Variant analysis is performed using the Bionano Access platform.

Abbreviations: WES, whole exome sequencing; ICLR, Illumina's Complete Long Reads; OGM, Optical Genome Mapping; SV, structural variant; SNV, single nucleotide variant; CNV, copy-number variant

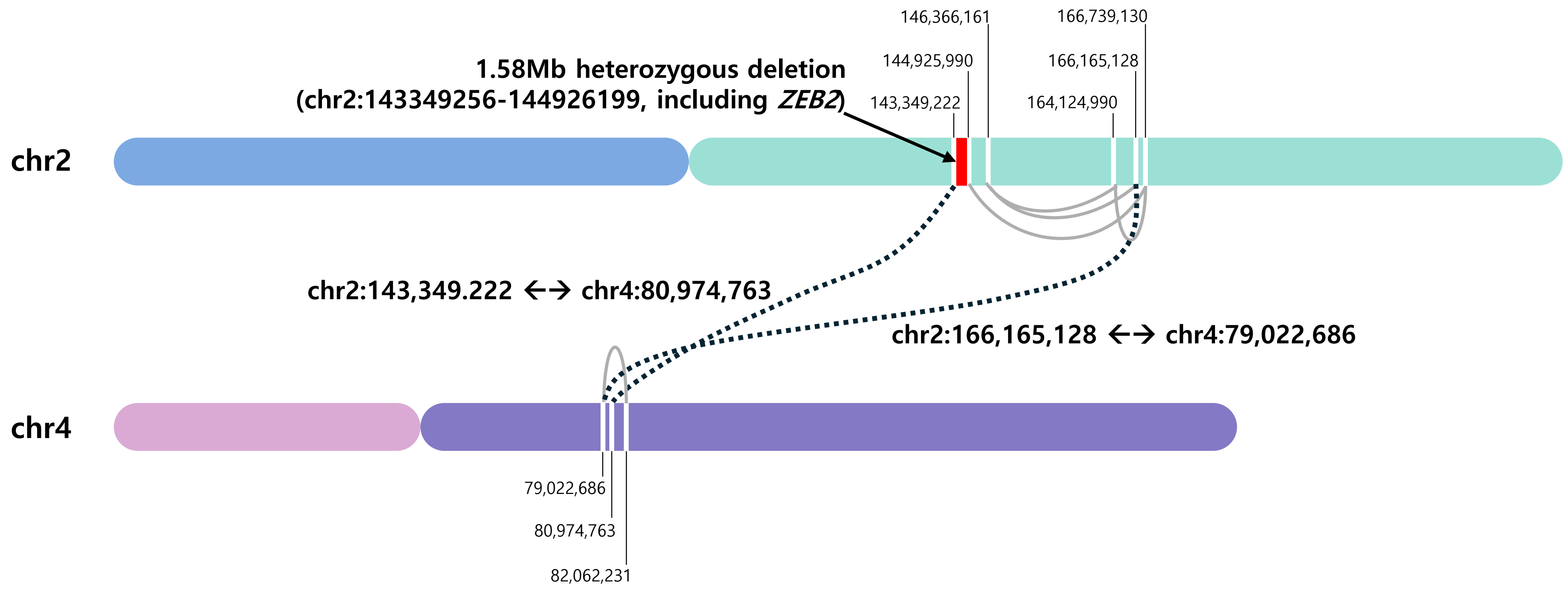

Supplemental Figure 2. Complex chromosomal rearrangements identified in Case 3 (GL00609P) involving a heterozygous 1.6 Mb deletion of the entire *ZEB2* gene. Rearrangement types and affected regions are schematically illustrated: gray lines represent intrachromosomal translocation, black dotted lines indicate interchromosomal translocations, and red shading marks deleted segments.

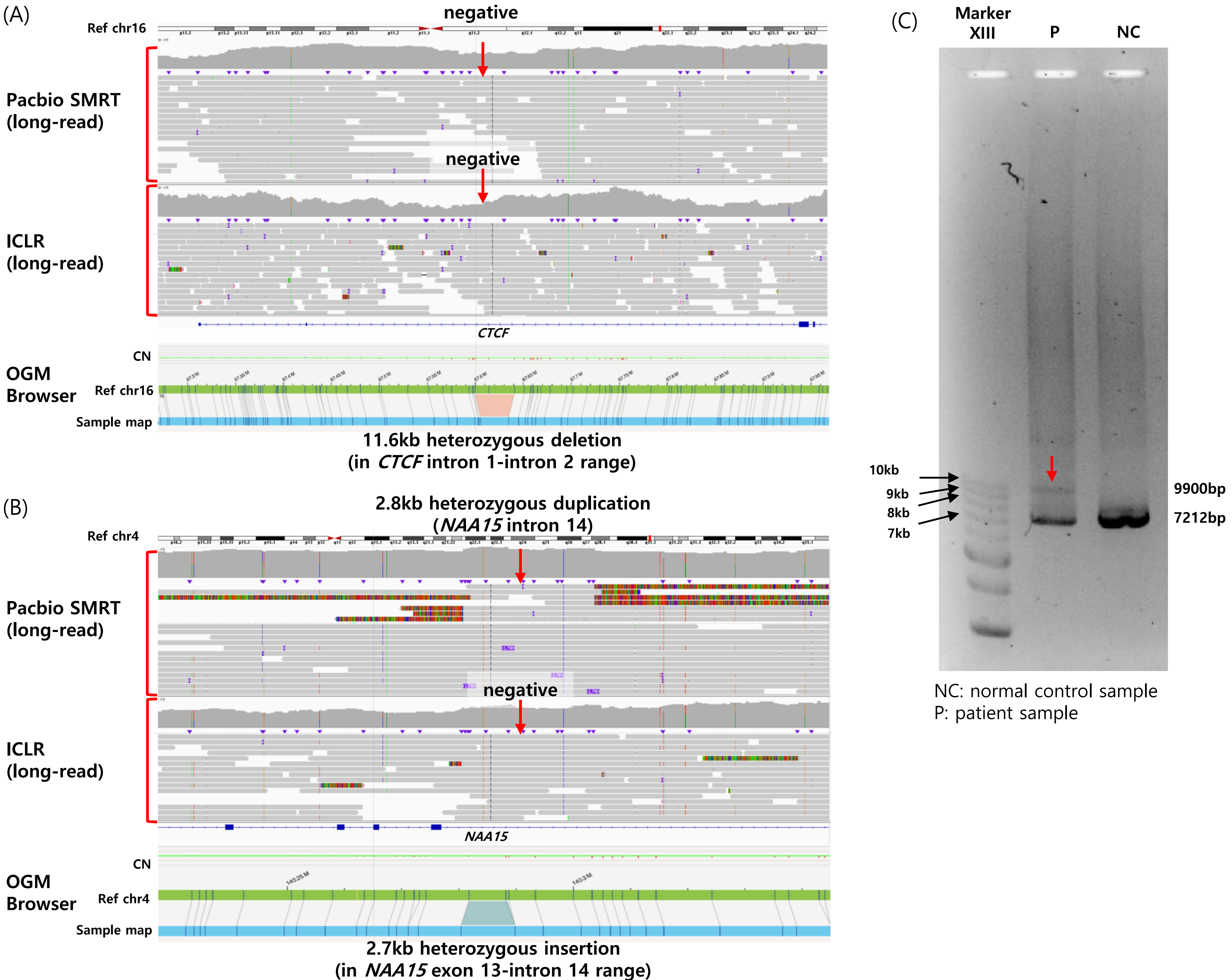
(A)
negative
Ref chr16
Pacbio SMRT
(long-read)
negative
ICLR
(long-read)
CTCF
OGM
Browser
CN
Ref chr16
Sample map
11.6kb heterozygous deletion
(in CTCF intron 1-intron 2 range)
(B)
2.8kb heterozygous duplication
(NAA15 intron 14)
Ref chr4
Pacbio SMRT
(long-read)
negative
ICLR
(long-read)
NAA15
OGM
Browser
CN
Ref chr4
Sample map
2.7kb heterozygous insertion
(in NAA15 exon 13-intron 14 range)
(C)
Marker
XIII
P
NC
10kb
9kb
8kb
7kb
9900bp
7212bp
NC: normal control sample
P: patient sample

Supplemental Figure 3. Experimental validation of OGM-only structural variant calls not detected by ICLR using Gap-PCR or PacBio sequencing.
(A) Case GL00612P: A heterozygous 11.6 kb deletion involving *CTCF* exon 2 was detected by OGM in a female patient with GDD and hypotonia. However, the deletion was not detected by ICLR, and PacBio SMRT long-read sequencing also yielded negative results, confirming the OGM call as a false positive.
(B) Case GL00536P: A heterozygous 2.7 kb insertion involving *NAA15* exons 13–14 was detected by OGM-only in a female patient with GDD, autistic behavior, and ADHD. This variant was not observed by ICLR; however, PacBio SMRT sequencing identified a 2.8 kb duplication within intron 14, flanked by AluS elements. Thus, the OGM call represented a true structural variant, but breakpoint resolution revealed no exon disruption, leading to classification as likely benign and exclusion from the patient's pathogenic findings.
(C) Gap-PCR validation of Case GL00536P showing a 9.9 kb allele containing the duplication in the patient. Compared with the 7.2 kb wild-type allele also observed in the negative control (NC), the difference in band size (~2.7 kb) confirms the presence of the duplication.

Abbreviation: ICLR, Illumina's Complete Long Reads; OGM, Optical Genome Mapping; CN, copy number; GDD, global developmental delay

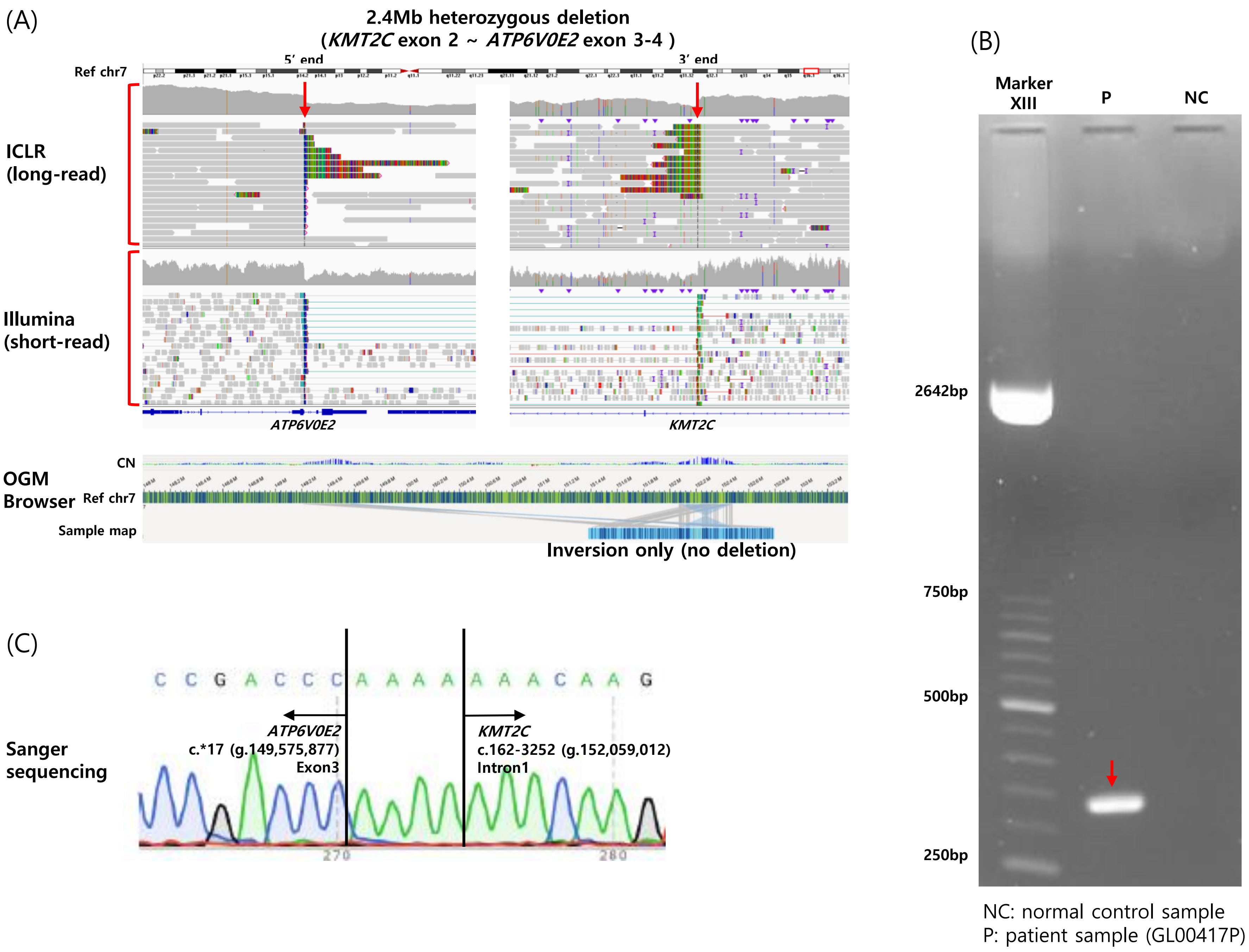
(A)
2.4Mb heterozygous deletion
(KMT2C exon 2 ~ ATP6V0E2 exon 3-4)
5' end
3' end
Ref chr7
ICLR
(long-read)
Illumina
(short-read)
ATP6V0E2
KMT2C
OGM
Browser
CN
Ref chr7
Sample map
Inversion only (no deletion)
(B)
Marker
XIII
P
NC
2642bp
750bp
500bp
250bp
NC: normal control sample
P: patient sample (GL00417P)
(C)
Sanger
sequencing
C C G A C C C A A A A A A A C A A G
ATP6V0E2
c.*17 (g.149,575,877)
Exon3
KMT2C
c.162-3252 (g.152,059,012)
Intron1
270
280

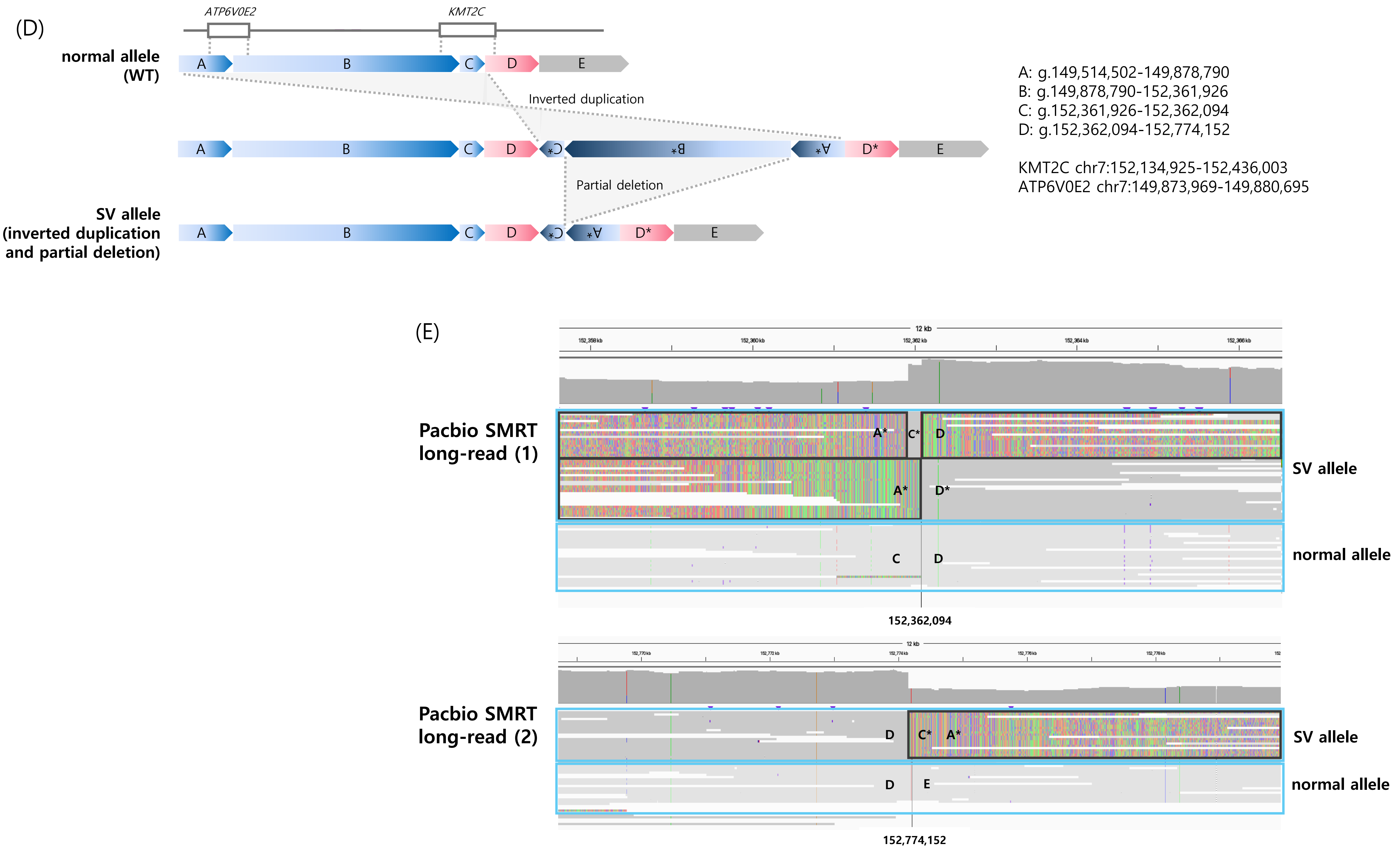

(D)
ATP6V0E2
KMT2C
normal allele (WT)
A B C D E
Inverted duplication
A B C D C* B* A* D* E
Partial deletion
SV allele (inverted duplication and partial deletion)
A B C D C* A* D* E
A: g.149,514,502-149,878,790
B: g.149,878,790-152,361,926
C: g.152,361,926-152,362,094
D: g.152,362,094-152,774,152
KMT2C chr7:152,134,925-152,436,003
ATP6V0E2 chr7:149,873,969-149,880,695
(E)
Pacbio SMRT long-read (1)
A* C* D
A* D*
SV allele
C D
normal allele
152,362,094
Pacbio SMRT long-read (2)
D C* A*
SV allele
D E
normal allele
152,774,152

(F)

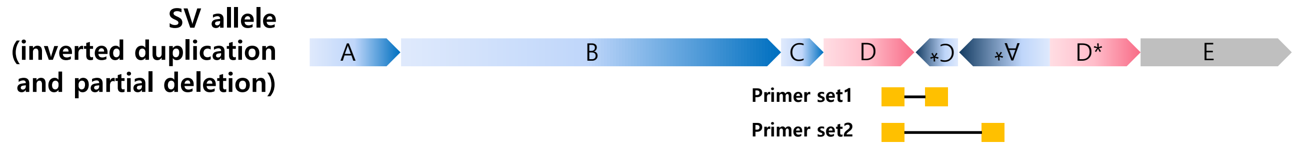

set	Forward sequence	Reverse sequence	product size	description
1	GGACAACACATTGAGCAAGCA	CACGATGGGTCCTCAGCTTT	269bp	Confirming Inverted duplication
2	GCTCACCATGCTTTGCTGTC	CCAACGAGTTCCAGAGCCTT	514bp	Confirming partial deletion in the inverted duplication area

(G)

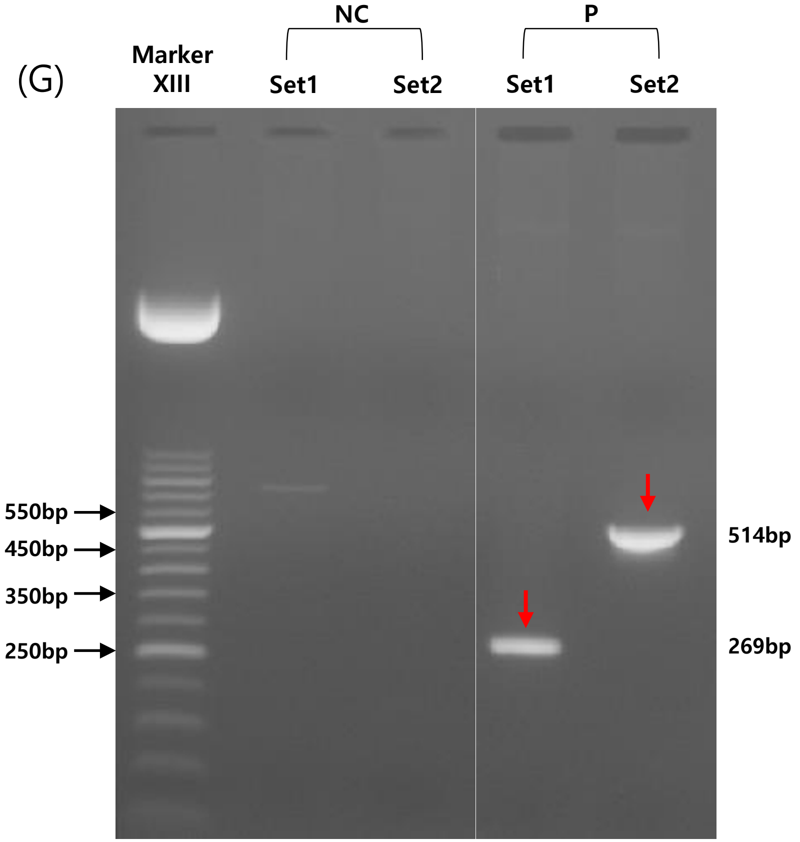

NC: normal control sample
P: patient sample (GL00417P)

Supplemental figure 4. Experimental validation of an ICLR-only structural variant call not detected by OGM using Gap-PCR or PacBio Sequencing: Case GL00417P

(A) ICLR identified a 2.4 Mb heterozygous deletion beginning at exon 2 of *KMT2C*, whereas OGM identified only an inversion in the same region without evidence of a deletion. (B) Gap-PCR analysis confirmed the presence of a deletion at the exact breakpoints identified by ICLR, producing a band in the patient sample not observed in the negative control. (C) Sanger sequencing of the Gap-PCR product validated the precise breakpoint junction between *ATP6V0E2* and *KMT2C.* (D) Further structural characterization by PacBio SMRT sequencing revealed that the apparent 2.4 Mb deletion was embedded within a more complex allele structure. Specifically, an inverted duplication encompassing the breakpoint region was identified, which also harbored a partial deletion. Genomic segments corresponding to this structure are indicated: Segment A, g.149,514,502–149,878,790; Segment B, g.149,878,790–152,361,926; Segment C, g.152,361,926–152,362,094; Segment D, g.152,362,094–152,774,152. The relevant gene coordinates are: *KMT2C*, chr7:152,134,925–152,436,003; *ATP6V0E2*, chr7:149,873,969–149,880,695.

(E) PacBio long-read sequencing enabled direct visualization of both the SV allele and the normal allele at the two breakpoints. The abnormal allele contained the inverted duplication with partial deletion, consistent with the structural model inferred in panel (D).

(F) Using two independent primer sets 1 and2, which targeting the predicted breakpoints, patient-specific bands of 269 bp and 514 bp were detected, whereas these bands were absent in the negative control. (G) Gap-PCR validation was performed using primer set 1 and 2 to confirm the structural model of the SV allele predicted by PacBio SMRT.
